# *Legionella pneumophila *induces cathepsin B-dependent necrotic cell death with releasing high mobility group box1 in macrophages

**DOI:** 10.1186/1465-9921-11-158

**Published:** 2010-11-22

**Authors:** Yoshitomo Morinaga, Katsunori Yanagihara, Shigeki Nakamura, Hiroo Hasegawa, Masafumi Seki, Koichi Izumikawa, Hiroshi Kakeya, Yoshihiro Yamamoto, Yasuaki Yamada, Shigeru Kohno, Shimeru Kamihira

**Affiliations:** 1 Department of Laboratory Medicine, Nagasaki University Graduate School of Biomedical Sciences, 1-7-1 Sakamoto, Nagasaki, 851-2128, Japan; 2 Second Department of Internal Medicine, Nagasaki University Graduate School of Biomedical Sciences, 1-7-1 Sakamoto, Nagasaki, 851-2128, Japan; 3 Global COE Program, Nagasaki University, 1-7-1 Sakamoto, Nagasaki, 851-2128, Japan

## Abstract

**Background:**

*Legionella pneumophila *(LPN) can cause a lethal infectious disease with a marked inflammatory response in humans. However, the mechanism of this severe inflammation remains poorly understood. Since necrosis is known to induce inflammation, we investigated whether LPN induces necrosis in macrophages. We also analyzed the involvement of lysosomal cathepsin B in LPN-induced cell death.

**Methods:**

The human monocytic cell line THP-1 was infected with LPN, NUL1 strain. MG132-treated cells were used as apoptotic control cells. After infection, the type of cell death was analyzed by using microscopy, LDH release and flow cytometry. As a proinflammatory mediator, high-mobility group box 1 (HMGB-1), was measured. Cathepsin B activity was also measured and the inhibitory effects of cathepsin B on LPN-induced cell death were analyzed.

**Results:**

THP-1 cells after treatment with high dose of LPN showed necrotic features with releasing HMGB-1. This necrosis and the HMGB-1 release were inhibited by a specific lysosomal cathepsin B inhibitor and were characterized by a rapid and high activation of cathepsin B that was not observed in apoptotic control cells. The necrosis was also accompanied by cathepsin B-dependent poly(ADP-ribose) polymerase (PARP) cleavage.

**Conclusions:**

We demonstrate here that *L. pneumophila *rapidly induces cathepsin B-dependent necrosis in a dose-dependent manner and releases a proinflammatory mediator, HMGB-1, from macrophages. This report describes a novel aspect of the pathogenesis of Legionnaires' disease and provides a possible therapeutic target for the regulation of inflammation.

## Introduction

*Legionella pneumophila *is an intracellular pathogen that causes rapidly advancing pneumonia and is sometimes life-threatening. After inhalation into the lung, the organism initially infects alveolar macrophages and replicates in these cells. The infected macrophages produce cytokines such as IL-β and TNF-α that activate both themselves and other immune cells [1]. However, although the functions of macrophages in response to this pathogen are crucial for innate immunity, the mechanism by which this pathogen induces such a severe immune response is not well understood.

In infectious diseases, cell death that occurs as a result of interactions between the infectious organism and the host cell can have important implications for host defense or bacterial survival. Apoptosis is a typical programmed cell death that is tightly regulated by various proteases, requires ATP and does not involve inflammation [2]. In contrast, necrosis, a type of cell death that is accompanied by inflammation, has been considered to represent accidental cell death due to exposure to supraphysiological conditions such as mechanical trauma, heat or cold [3]. During interactions between pathogens such as *Shigella *[4], *Salmonella *[5] and *Mycobacterium tuberculosis *[6] and the host immune response, there have been some reports of cell death induced by these bacteria that appears to have features of necrosis. While *L. pneumophila *has been shown to induce apoptosis in macrophages or monocytic cell lines when the cells were infected at a low dose of bacteria [7-9], induction of apoptosis is not necessarily associated with pathogenesis in severe infections. Thus, necrosis can contribute to inflammation in Legionnaires' disease, although there are few reports concerning the induction of necrosis by *L. pneumophila*, in which a high dose of bacteria was used [10,11].

Recent research has implicated lysosomal function in cell death [12]. Many types of proteases and chemical agents that are known apoptosis inducers, such as caspases, anticancer agents and reactive oxygen species, may also be involved in cell death via the modulation of lysosomal membrane permeability, and some of these agents also induce necrosis [13]. Similarly, it has been shown that necrosis, like apoptosis, can be regulated by intracellular molecules, and lysosomes in particular are considered to be important organelles for programmed necrosis [13,14].

In this report, we determined if *L. pneumophila *induces necrotic cell death in a monocytic cell line and in murine macrophages by comparing cell death induced by *L. pneumophila *with that induced by an apoptotic agent. We also examined the role of lysosomal enzymes in *L. pneumophila*-induced cell death. We found that potent activation of cathepsin B leads to necrosis accompanied by inflammation in cells infected with a high dose of *L. pneumophila*. In addition, cell death and inflammation were inhibited by attenuation of cathepsin B.

## Materials and methods

### Reagents

PARP antibody was from Cell Signaling Technology (Danvers, MA) and anti-cathepsin B antibody (CA10) was from Abcam (Cambridge, MA). CA074Me and zVADfmk were obtained from the Peptide institute (Osaka, Japan).

### Bacterial strains

The *L. pneumophila *NUL1 bacterial strain, serogroup 1, which was clinically isolated from the sputum of a patient at Nagasaki University Hospital [15], was used. The bacteria were cultured on buffered charcoal yeast extract α agar plates for 3 days. The bacteria were stored at -80°C in a Microbank system (Pro-Lab Diagnostics, Ontario, Canada) until use.

### Animals

Female A/J mice, 6-8-weeks old, were purchased from Japan SLC, Inc. (Shizuoka, Japan). All animals were housed in a pathogen-free environment and received sterile food and water in the Laboratory Animal Center for Biomedical Science at Nagasaki University. Animal care and experimental procedures were performed in accordance with the Guidelines for Animal Experimentation of Nagasaki University with approval of the Institutional Animal Care and Use Committee.

### Cell lines and treatment procedure

The human monocytic cell line THP-1 was cultured in RPMI1640 medium containing 10% FBS. Murine alveolar or peritoneal macrophages were obtained from bronchoalveolar lavage fluid or peritoneal lavage fluid 3 days after injection of 4% thioglycollate into the peritoneum, respectively. The cells were plated into 96-well plates at a density of 1 × 10^5 ^cells/well for all experiments except for fluorescent microscopy. After addition of *L. pneumophila *or MG132, the plate was centrifuged at 1000 × *g *for 5 min. For inhibition studies, the cells were pre-treated with CA074Me for 30 min before stimulation.

### Cytotoxicity assay

Cell death was evaluated using a cytotoxicity detection kit (Roche Diagnostics GmbH, Mannheim, Germany) based on the measurement of LDH according to the manufacturer's instructions. To determine cytotoxicity, the value of samples from 1% Triton-X-treated cells was defined as 100% cell death.

### Optical microscopy

Cells were stained with 0.2% trypan blue and trypan blue-positive and -negative cells were counted. To observe morphological findings, the cells were centrifuged at 1,000 × *g *for 1 min, fixed onto slides and were then stained with Diff-Quik (Sysmex, Hyogo, Japan). Cellular morphological features were categorized by two cytologists. One hundred cells were counted three times and the results are presented as an average value.

### Fluorescent microscopy

Mice macrophages were plated into a CultureWell chambered coverglass (Life Technologies, Carlsbad, CA) at a density of 1 × 10^3 ^cells/well (alveolar macrophages) or 1 × 10^5 ^cells/well (peritoneal macrophages) and were cultured in fresh medium overnight. After treatment, the cells were incubated with PBS containing 7.5 μg/mL Hoechst 33342 and 2 μg/mL PI for 30 min. The preparation was observed using a Leica DM400B fluorescent microscope (Leica Microsystems GmbH, Wetzlar, Germany).

### Cathepsin B activity

Cells were washed and homogenized at 4°C in lysis buffer as described previously [16]. Cell lysates (10 μg) were incubated with 20 μM z-Arg-Arg-MCA (Peptide Institute. Inc., Osaka, Japan) at 37°C for 2 h and the release of amino-4-methylcoumarin (AMC) was monitored using a SPECTRA Flour microplate reader (Tecan Japan Co., Ltd., Kanagawa, Japan). All experiments were performed in duplicate.

### Flow cytometry

To evaluate cell death changes, cells were simultaneously stained with FITC-conjugated Annexin-V and the non-vital dye PI (Bender Medsystems, Vienna, Austria) to enable discrimination of intact cells (Annexin-V^-^, PI^-^), early apoptotic cells (Annexin-V^+^,PI^-^) and late apoptotic or necrotic cells (Annexin-V^+^, PI^+^). Cells were harvested after treatment, and 10^4 ^cells per sample were analyzed using a FACSCalibur flow cytometer and CellQuest software (BD Biosciences Immunocytometry Systems, San Jose, CA).

### Western blotting

Cells were harvested after treatment, and were washed and homogenized at 4°C in RIPA buffer. Cell lysates (20-50 μg) were resolved by electrophoresis on a 12.5% polyacrylamide gel, and were transferred to a polyvinylidine difluoride membrane. After blocking the membrane in 10% FBS and 0.1% Tween-20 in Tris-buffered saline for 1 h at room temperature, blots were hybridized with primary antibodies overnight at 4°C. After hybridization with a secondary antibody, the immunocomplexes were visualized using an ECL Western Blotting Detection System (GE Healthcare, Chalfont St. Giles, UK).

### HMGB-1 concentration

The concentration of extracellular HMGB-1 was measured using an HMGB-1 ELISA Kit II (Shino Test, Kanagawa, Japan) according to the manufacturer's instructions. Briefly, 10 μL of samples were added to an anti-HMBG-1 antibody-coated plate. After incubation at 37°C for 24 h, the plate was incubated with peroxidase-conjugated anti-HMGB-1 monoclonal antibody for 2 h at room temperature. Color was developed using a 3,3',5,5'-tetramethylbenzidine and peroxidase solution. Absorbance was read at 450 nm.

### Statistical analysis

Cytotoxicity, morphological classification and HMGB-1 concentrations were expressed as means ± SD. One-way analysis of variance was used to determine statistically significant differences between groups. Tukey's test was used to confirm differences between individual groups.

## Results

### A High dose of *L. pneumophila *induces necrotic cell death

**Figure 1:**
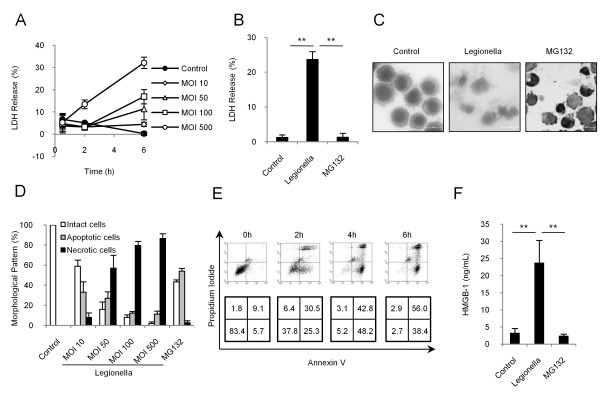
**A high dose of *L. pneumophila *induces necrotic cell death in THP-1 cells**. (A) Cytotoxicity of *L. pneumophila *was evaluated by assay of LDH release from THP-1 cells. (B) LDH release after 6h-stimulation with an MOI 500 of *L. pneumophila *or 10 μM of the apoptotic inducer MG132. (C) Diff-Quik staining after 6h-stimulation with an MOI 500 of *L. pneumophila *or MG132. *L. pneumophila *induced necrotic features such as loss of plasma membrane integrity and irregular and poorly-stained nuclei. The scale bar represents 20 μm. (D) Microscopic findings of the three cell groups identified following 6 h stimulation. *L. pneumophila *increased the percentage of necrotic cells in a dose-dependent manner. In contrast, MG132 induced mainly apoptotic cells. (E) Flow cytometric analysis of THP-1 cells, infected with an MOI of 500 of *L. pneumophila*, and stained with Annexin-V and PI. The number at the bottom of each panel indicates the percentage of cells detected in that area. (F) Extracellular HMGB-1 after 6 h incubation following infection with an MOI of 500 of *L. pneumophila*, or with MG132. HMGB-1 was significantly increased by *L. pneumophila *but not by MG132. Values are expressed as means ± SD. ** indicates p < 0.01.

The levels of *L. pneumophila *used were calculated based on the ratio of bacteria to cells, and are expressed as multiplicity of infection (MOI). First, we assayed the time course of lactate dehydrogenase (LDH) release into the culture medium following incubation of THP-1 cells with various levels of *L. pneumophila *(Figure 1A). *L. pneumophila *increased the levels of LDH, as well as the percentage of trypan blue-positive cells (data not shown), in a dose- and time-dependent manner but not an apoptosis inducer, MG132 [17] (Figure 1B). Following 6 h of incubation with an MOI 500 of *L. pneumophila*, the cells showed characteristic features that are consistent with necrosis such as the loss of plasma membrane integrity and irregular and poorly-stained nuclei in contrast to MG132-treated apoptotic cells (Figure 1C). The necrotic cells partially appeared even when cells were infected by a low dose of bacteria, and the necrotic cells increased in a dose-dependent manner with addition of *L. pneumophila *(Figure 1D). Thus, an MOI 500 of bacteria was used in subsequent experiments to analyze the character of *L. pneumophila*-induced necrotic cells. In flow cytometry analysis with Annexin-V and PI binding, *L. pneumophila*-treated cells were divided into two clusters over time of treatment (Figure 1E). The cells in one group rapidly transformed into cells that were double-positive for Annexin-V and PI that represented 30.5% of the total population at 2 h. The second group of cells began to appear as Annexin-V-positive and PI-negative cells at 2 h and some of these cells were observed as double-positive cells at 6 h. High mobility group box 1 (HMGB-1), known as a necrotic marker as well as a proinflammatory molecule, was also significantly increased in the culture medium after *L. pneumophila *stimulation (Figure 1F). These suggest that a high dose of *L. pneumophila *induces necrotic cell death.

### *L. pneumophila*-induced necrotic cell death is dependent on cathepsin B

**Figure 2:**
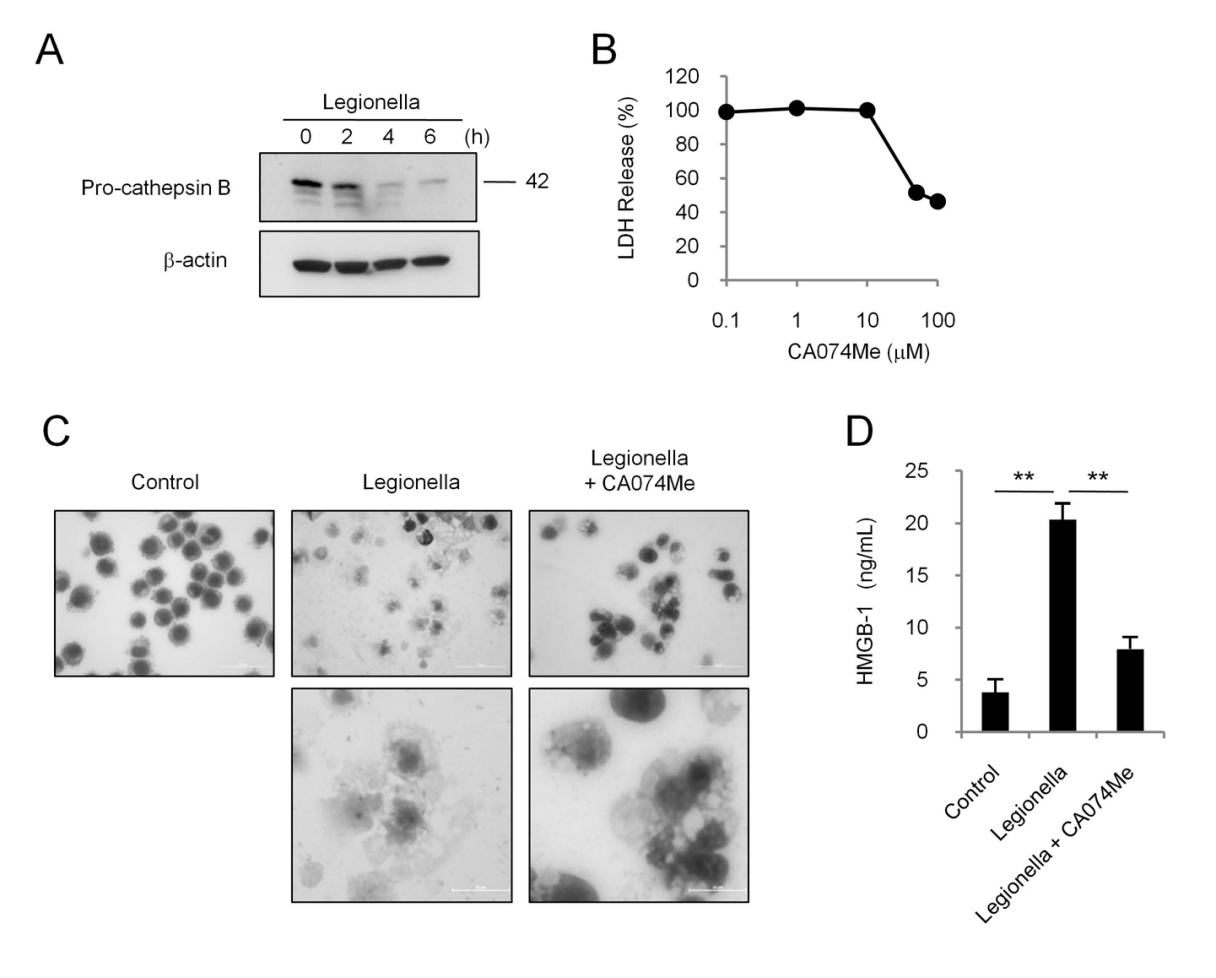
***L. pneumophila*-induced THP-1 cell death is cathepsin B-dependent**. (A) Western blot analysis of pro-cathepsin B expression following stimulation with an MOI of 500 of *L. pneumonia *for the indicated times. (B) The effect of the specific cathepsin B inhibitor, CA074Me, on the *L. pneumophila *-induced LDH. CA074Me inhibited *L. pneumophila*-induced cytotoxicity at concentrations greater than 50 μM. (C) The microscopic findings after pretreatment with CA074Me (100 μM) on *L. pneumophila*-induced necrotic cells. The top scale bar represents 50 μm and the bottom represents 20 μm. (D) The effect of CA074 pretreatment on *L. pneumophila-*induced elevation of HMGB-1. Error bars in (B) and (D) represent means ± SD. ** indicates p < 0.01.

To examine the involvement of cathepsin B in *L. pneumophila*-induced necrotic cell death, we assayed the cellular level of pro-cathepsin B. The level of pro-cathepsin B gradually diminished over time following infection, suggesting its processing into an active enzyme (Figure 2A). Pre-treatment with a specific cathepsin B inhibitor, CA074Me, at a concentration of 50 or 100 μM decreased *L. pneumophila-*induced LDH by about 50% (Figure 2B). Thus, we used CA074Me at a concentration of 100 μM in subsequent inhibitory experiments. This concentration of CA074Me alone did not affect all of the parameters in this study and bacterial growth. Microscopic observation clearly indicated fewer necrotic cells after CA074Me pretreatment than non-treated cells (Figure 2C). Instead, the number of intact cells, which had maintained a plasma membrane and a normally stained and shaped nucleus, and apoptotic cells seemed to increase. The extracellular HMGB-1 was also significantly suppressed by CA074Me pretreatment compared to *L. pneumophila *stimulation alone (Figure 2D). These findings were also observed in the assay with another isolate (data not shown). These suggest that *L. pneumophila*-induced necrotic cell death is dependent on cathepsin B.

### Cathepsin B regulates both necrotic and apoptotic cell death

**Figure 3:**
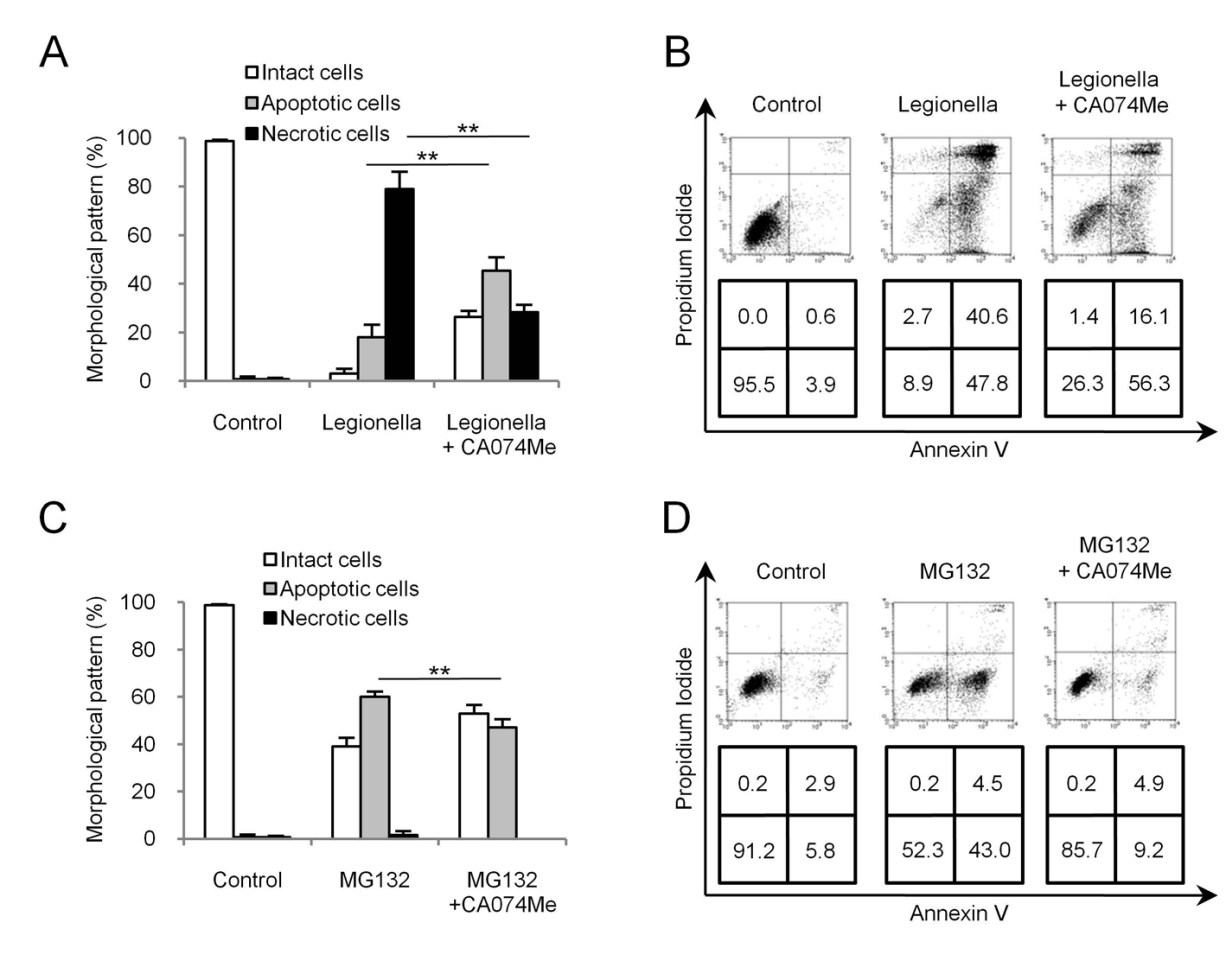
**Cathepsin B regulates necrosis and apoptosis**. (A) Classification of *L. pneumophila*-infected cells 6 h after stimulation based on morphological features. The percentage of necrotic cells was significantly decreased by pretreatment with CA074. (B) Annexin V and PI stained cells were evaluated by flow cytometry analysis 6 h after stimulation with *L. pneumophila*. The percentage of stained cells is indicated at the bottom of each panel. (C) Morphological classification of MG132-treated cells. MG132 induced-apoptotic cells were inhibited by pretreatment with CA074Me. (D) Flow cytometric analysis of MG132-treated cells stained with Annexin V and PI at 6 h. Values are expressed as means ± SD. ** indicates p < 0.01.

Next, the effect of CA074Me on the type of cell was analyzed. CA074Me pretreatment decreased the percentage of necrotic cells to 50% or lower than that of non-treated cells (Figure 3A). Annexin-V- and PI-double-positive cells decreased and that the intact double-negative cells increased (Figure 3B). In MG132-treated cells, CA074Me pretreatment slightly suppressed the percentage of apoptotic cells (Figure 3C) and this effect was more clearly observed in flow cytometry (Figure 3D). These results indicated that cathepsin B is involved in the induction of both necrotic and apoptotic cell death.

### *L. pneumophila*-induced high activation of cathepsin B is involved in poly(ADP-ribose) polymerase (PARP) cleavage and cell death

**Figure 4:**
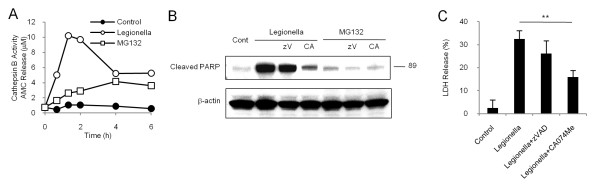
***L. pneumophila*-induced high activation of cathepsin B is involved in PARP cleavage and cell death**. (A) The activity of cathepsin B in cell lysates after *L. pneumophila *(MOI of 500) or MG132 treatment was measured. *L. pneumophila *rapidly and strongly enhanced cathepsin B activation. In contrast, MG132 gradually activated cathepsin B over the time of assay. Data are representative of three independent experiments. (B) Effects of CA074Me and a pan-caspase inhibitor (zVADfmk, 100 μM) on PARP cleavage after *L. pneumophila *or MG132 treatment at 2 h, assessed by western blot. *L. pneumophila*-induced PARP cleavage was attenuated by CA074Me but not by zVADfmk. In contrast, zVADfmk showed mild inhibition of MG132-induced PARP cleavage. Cont; control, zV; zVADfmk, CA; CA074Me. (C) Effects of CA074Me and zVADfmk on the cytotoxicity of *L. pneumophila*-treated cells. CA074Me, but not zVADfmk, significantly decreased *L. pneumophila*-induced LDH. Values are expressed as means ± SD. ** indicates p < 0.01.

To analyze the different functions of cathepsin B on cell death, we examined the time course of cathepsin B activation. Interestingly, *L. pneumophila *induced a rapid peak of cathepsin B activity that occurred within 2 h and subsequently dropped to approximately half of the peak value, whereas MG132 treatment induced a gradual elevation (Figure 4A). The high cathepsin B activation was also observed in other experiments within 6 h. CA074Me almost completely inhibited the activation of cathepsin B (data not shown). Next, we examined the cleavage of PARP at 2 h with using CA074ME or a pan-caspase inhibitor, zVADfmk (Figure 4B) because PARP cleavage can be observed in necrosis [18] and apoptosis [19]. *L. pneumophila *dramatically enhanced the intensity of cleaved PARP compared to MG132 and this enhancement was attenuated more strongly by CA074Me than by zVADfmk. *L. pneumophila*-induced LDH was also significantly suppressed by pretreatment with CA074Me but not by pretreatment with zVADfmk (Figure 3C). These results suggest that *L. pneumophila *induces caspase-independent but cathepsin B-dependent PARP cleavage and cell death.

### Cathepsin B is involved in *L. pneumophila*-induced murine macrophage death

**Figure 5:**
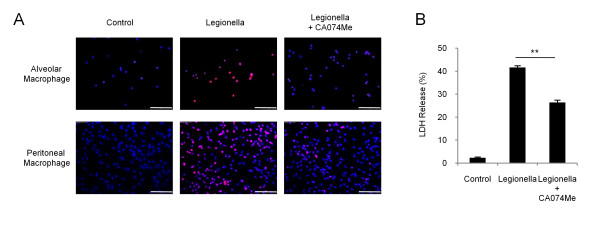
**Cathepsin B is involved in *L. pneumophila*-induced murine macrophage death **(A) Macrophages derived from the bronchoalveolar or peritoneal lavage fluid of A/J mice were stimulated with an MOI 500 of *L. pneumophila *for 6 h followed by staining with Hoechst 33342 and PI. The PI-positive cells (red) represent necrotic cells. *L. pneumophila *infection increased, and pretreatment with CA074Me decreased, the number of PI-positive cells. The scale bar represents 75 μm. (B) LDH release 6 h after *L. pneumophil*a-infection in peritoneal macrophages. CA074Me pretreatment had an inhibitory effect on *L. pneumophila*-induced cytotoxicity. Values are expressed as means ± SD. ** indicates p < 0.01.

We examined whether the cathepsin B-mediated, *L. pneumophila*-induced cell death also occurs in murine macrophages. A/J mouse alveolar or peritoneal macrophages were stained with PI, which serves as a marker of plasma membrane permeability (Figure 5A). Most of the alveolar macrophages were PI-positive after stimulation with *L. pneumophila*. However, pretreatment with CA074Me markedly reduced the number of PI-positive cells. In the assay of peritoneal macrophages, the LDH level that was elevated by *L. pneumophila *was decreased by pretreatment with CA074Me (Figure 5B).

## Discussion

In this paper, we studied cell death induced by *L. pneumophila *in macrophages and compared this death with that induced by an apoptotic agent. A high dose of *L. pneumophila *induced necrotic cell death in which cathepsin B played an important role in determination of the mechanism of cell death. This study reveals a novel aspect of the pathogenesis of Legionnaires' disease and identifies potential novel therapeutic targets for the regulation of inflammation.

Research regarding the mechanism of cell death related to *L. pneumophila *infection has focused mainly on apoptosis induced in monocytic cell lines or in murine macrophages with using a low dose of bacteria, such as an MOI 50 or lower [7,9]. On the other hand, Husmann and Johnson reported that infection with high multiplicity of *L. pneumophila *induce cytotoxicity in guinea pig peritoneal macrophages [20]. Similarly, the same dose in this study also induced necrotic cell death in mouse bone-marrow derived macrophages [11]. However, mechanisms of necrotic cell death were not determined.

Apoptosis is generally recognized as a non-inflammation-producing cell death [3], however, serious inflammation is usually observed in patients with Legionnaires' disease. Since necrosis is known to induce inflammation, necrosis could potentially be involved in the pathogenesis of Legionnaires' disease. Necrotic cells appeared even after the treatment with a low dose of bacteria as shown in Figure 1D, and necrotic cells increased HMGB-1. Thus, the mixed type of cell death can be involved in the pathogenesis of Legionnaires' disease.

Apoptosis is a major programmed cell death and its signaling pathways and inducers have been well studied [21,22]. There is some evidence that necrosis, like apoptosis, is regulated by several enzymes [23] such as lysosomal enzymes, cathepsins [24]. For instance, cathepsin B mediates apoptosis induced by TRAIL in oral cancer cells [25] and necrosis induced by nigericin in THP-1 cells [26]. In this study, cathepsin B also regulated both apoptotic and necrotic cell death.

Thus, cathepsin B is a non-caspase protease that can lead to apoptosis and necrosis. However, the mechanistic details of cathepsin B involvement in each type of cell death are still uncertain. The type of cell death mediated by lysosomal enzymes appears to depend on the magnitude of lysosomal permeabilization and the consequent release of lysosomal enzymes into the cytosol. Thus, the massive release of enzymes after complete lysosomal permeabilization induces necrotic cell death, whereas partial permeabilization with the release of lower levels of enzymes leads to apoptotic cell death [14]. *L. pneumophila*-induced high cathepsin B activity would support the possibility that complete lysosomal permeabilization had occurred, resulting in necrotic cell death. However, as pretreatment with CA074Me showed incomplete inhibition of cell death, reactive oxygen species [27] and multiple proteases such as calpains [28] and other cathepsins [29,30] may be involved in *L. pneumophila*-induced necrotic cell death by cross-talking.

Recently, several cell death pathways have been newly identified. Pyroptosis is a caspase-1-dependent cell death and results in releasing inflammatory cytokines such as IL-1β and IL-18 [31]. It is reported that *L. pneumophila *induces pyroptosis [32,33]. Pyroptosis features plasma membrane permeability and nuclear condensation, however, cells in this study showed poor-stained nuclei which is mismatched to pyroptosis. In addition, in prescreening of inhibitory effect of cell death in this study, a specific caspase-1 inhibitor, zYVADcmk, had no effect on the LDH release (data not shown). Necroptosis or programmed necrosis, another type of cell death, is a necrotic cell death which is executed by enzymatic regulation such as receptor-interacting protein kinase 1, calpains and cathepsins [23]. The cell death in this study showed necrotic feature and cytotoxicity induced by *L. pneumophila *was independent of caspases including caspase-1 but dependent of cathepsin B, suggesting that the cell death in this study was consistent with necroptosis rather than pyroptosis. However, our results with the immortalized cell line may need to be interpreted with caution in terms of cell death phenotypes as the immortalization process can affect cell death pathways.

In the cell death in the host response to *L. pneumophila *infection, flagellin is an important bacterial component as an inducer of pyroptosis [32]. Pyroptosis is also observed during *Salmonella *[34] or *Shigella *[35] infection, however, roles of bacterial flagellin on cell death pathways are different in these bacteria. Since we did not rule out the possibility of the involvement of flagellin and other components, we need further examination to reveal the relationship between bacterial components and cell death.

Cleaved PARP has been widely employed as a useful hallmark of apoptosis because PARP is processed by caspases [36]. However, *L. pneumophila *induced necrotic cell death in this study was accompanied by cleavage of PARP. This conflicting result can be attributed to excessive cathepsin B activation because PARP can be processed not only by caspases but also by cathepsin B and other proteases such as calpain, granzyme B and cathepsin G [18,37]. Indeed, *L. pneumophila*-induced cleaved PARP was attenuated by a cathepsin B inhibitor but not by a pan-caspase inhibitor, and the necrotic features of the cells were probably caused by indiscriminate degradation of cellular components by cathepsin B.

For the treatment of serious infectious diseases, it is important to regulate excessive inflammation. HMGB-1 is a cytokine that mediates strong systemic inflammatory responses, activates prototypical inflammatory responses in immune cells [38], and clinically causes acute lung injury [39]. Sepsis is a major example of severe inflammatory disease in which a vicious cycle involving HMGB-1 is implicated and elevated serum levels of HMGB-1 correlate with a worse prognosis in patients with sepsis [40]. Because severe cases of Legionnaires' disease can occur against which antibiotic therapy alone is insufficient even using antibiotics with high antibacterial activities [41], this excessive inflammation involving HMGB-1 induced by *L. pneumophila *is associated with the severity of the pathogenesis. Although further examination is necessary to determine whether inhibition of necrosis is actually beneficial for the treatment of Legionnaires' disease, the programmed necrosis induced by *L. pneumophila *that is described in this study may provide new information for the identification of therapeutic targets.

In conclusion, we report that a high dose of *L. pneumophila *induces cathepsin B-dependent necrosis in macrophages and that HMGB-1 release was also regulated by inhibition of necrotic cell death. The pattern of cathepsin B activation was important for subsequent PARP cleavage and the type of cell death. Necrotic cell death can contribute to the pathogenesis of Legionnaires' disease and its severity and regulation of this cell death may provide some benefit in treatment of this disease.

## Abbreviations

(HMGB-1): high-mobility group box 1; (PARP): poly(ADP-ribose) polymerase; (LDH): lactate dehydrogenase.

## Competing interests

The authors declare that they have no competing interests.

## Authors' contributions

YM designed the study, carried out the molecular studies, the cell culture, the animal study, image analysis and data analysis, participated in the flow cytometry and drafted the manuscript. HH carried out the flow cytometry. KY, HH, YYamad and SKa participated in the design of the study and revised the manuscript. SN, MS, KI, HK, YYamam and SKo coordinated the study. All authors read and approved the final manuscript.
